# Chronic Toll-like receptor 4 stimulation in skin induces inflammation, macrophage activation, transforming growth factor beta signature gene expression, and fibrosis

**DOI:** 10.1186/ar4598

**Published:** 2014-07-01

**Authors:** Giuseppina Stifano, Alsya J Affandi, Allison L Mathes, Lisa M Rice, Sashidhar Nakerakanti, Banafsheh Nazari, Jungeun Lee, Romy B Christmann, Robert Lafyatis

**Affiliations:** 1 Boston University School of Medicine, E501, Arthritis Center, 72 East Concord Street, Boston, MA 02118-2526, USA; 2 Department of Rheumatology & Clinical Immunology, Laboratory of Translational Immunology, University Medical Center Utrecht, Heidelberglaan 100, Utrecht, CX 3584, The Netherlands

## Abstract

**Introduction:**

The crucial role of innate immunity in the pathogenesis of systemic sclerosis (SSc) is well established, and in the past few years the hypothesis that Toll-like receptor 4 (TLR4) activation induced by endogenous ligands is involved in fibrogenesis has been supported by several studies on skin, liver, and kidney fibrosis. These findings suggest that TLR4 activation can enhance transforming growth factor beta (TGF-β) signaling, providing a potential mechanism for TLR4/Myeloid differentiation factor 88 (MyD88)-dependent fibrosis.

**Methods:**

The expression of *TLR4*, *CD14* and *MD2* genes was analyzed by real-time polymerase chain reaction from skin biopsies of 24 patients with diffuse cutaneous SSc. In order to investigate the effects of the chronic skin exposure to endotoxin (Lipopolysaccharide (LPS)) *in vivo* we examined the expression of inflammation, TGF-β signaling and cellular markers genes by nanostring. We also identified cellular subsets by immunohistochemistry and flow cytometry.

**Results:**

We found that TLR4 and its co-receptors, MD2 and CD14, are over-expressed in lesional skin from patients with diffuse cutaneous SSc, and correlate significantly with progressive or regressive skin disease as assessed by the Delta Modified Rodnan Skin Score. *In vivo*, a model of chronic dermal LPS exposure showed overexpression of proinflammatory chemokines, recruitment and activation of macrophages, and upregulation of TGF-β signature genes.

**Conclusions:**

We delineated the role of MyD88 as necessary for the induction not only for the early phase of inflammation, but also for pro-fibrotic gene expression via activation of macrophages. Chronic LPS exposure might be a model of early stage of SSc when inflammation and macrophage activation are important pathological features of the disease, supporting a role for innate immune activation in SSc skin fibrosis.

## Introduction

Systemic sclerosis (SSc) is a chronic connective tissue disease of unknown etiology, characterized by heterogeneous clinical manifestations and an unpredictable course. Hallmarks of this disease are inflammation, autoimmunity, vascular damage, and fibrosis [1]. Fibrosis appears most likely induced by inappropriate production of transforming growth factor-β (TGF-β), or other pro-fibrotic cytokines, such as connective tissue growth factor (CTGF), IL-13 and thymic stromal lymphopoietin (TSLP), inducing excessive accumulation of extracellular matrix (ECM) components by activated fibroblasts [2-4]. Although the stimulus for production of pro-fibrotic cytokines in SSc remains uncertain, these cytokines might be released or activated by infiltrating immune cells that are most prominent during the inflammatory stage of SSc [2,3,5,6]. The importance of immune-mediated pathogenesis of SSc is supported by several observations, including the close relationship in clinical features and genetic associations with systemic lupus erythematosus (SLE), where inflammation appears to be induced or amplified by activation of Toll-like receptors (TLRs) [7].

TLRs are type I transmembrane proteins with extracytoplasmic domains (ectodomains) responsible for ligand binding, and intracellular Toll-interleukin 1 (IL-1) receptor (TIR) domains necessary for downstream signal transduction. Each TLR can recognize specific microbial components, known as pathogen associated molecular patterns (PAMPs). Some TLRs (TLR2, TLR4, TLR5, and TLR6) are expressed on the cellular surface, and others (TLR3, TLR7, TLR8, and TLR9) are normally located in intracellular compartments [8,9].

TLR4, one of the first TLRs identified, was recognized as the receptor able to respond to bacterial lipopolysaccharide (LPS), a component of the outer membrane of gram-negative bacteria, causing septic shock [10]. On the cell surface TLR4 forms a complex with myeloid differentiation factor-2 (MD2) acting as the main LPS-binding component [11]. Additional proteins, such as LPS-binding protein (LBP), a soluble plasma protein, and CD14 are also involved in LPS binding [12]. CD14, a glycosyl-phosphatidylinositol (GPI)-anchored protein without transmembrane and intracellular domains, is expressed on the cellular surface (mCD14) or produced in a soluble form (sCD14). CD14 enhances LPS responsiveness by binding LPS and facilitating LPS transfer to TLR4/MD-2 [13]. It is primarily expressed on cells of the monocyte/macrophage lineage, but is also expressed at low levels on neutrophils, non-myeloid cells, B-lymphocytes, endothelial cells and mammary epithelial cells. Cells that do not express mCD14 can use sCD14 to recognize LPS [14,15]. After the binding of LPS to TLR4, signaling transduction follows two different pathways: a Myeloid differentiation factor 88 (MyD88)-dependent pathway activated by all TLRs except TLR3, and a MyD88-independent pathway requiring the TIR-containing adaptor molecule (TICAM), also used by TLR3. Both pathways trigger downstream signaling cascades inducing the production of proinflammatory cytokines, chemokines, and type I interferon [8].

Interest in TLR4 as a pro-fibrotic mediator has been stimulated by the observation that endogenous molecules can interact directly or indirectly with TLR4, such as connective tissue molecules (hyluronan, fibrinogen, alternatively spliced fibronectin extradomain A (Fn-EDA), tenascin C, and biglycan), heat-shock proteins (Hsp60, Hsp70, Gp69), cellular stress protein (high mobility group box 1 protein (HMGB1), beta-defensin 2, heparan sulfate, and surfactant protein-A [16]. In the past few years, the hypothesis that TLR4 activation induced by endogenous ligands is involved in fibrogenesis has been supported by several studies on skin, liver, and kidney fibrosis [17-19]. These studies have led to the notion that damage associated molecular patterns (DAMPs) from these or other proteins may become exposed during inflammation, causing or perpetuating inflammation and fibrosis. Other studies have suggested that TLR4 can directly enhance TGF-β signaling [17,20], providing a potential mechanism for TLR4-mediated fibrosis.

In the present study, we sought to better understand the role of TLR4 activation in SSc, by investigating the clinical correlations between the expression of TLR4 and its co-receptors, MD2 and CD14, in the skin of SSc patients, and by investigating in vivo the effect of chronic cutaneous LPS exposure.

## Materials and methods

### Study participants

The Boston University Medical Center Institutional Review Board (Boston, MA, USA) reviewed and approved the conduct of this study. Informed consent was obtained from all patients and healthy subjects (diffuse cutaneous SSc (dcSSc) (n = 24) according to diagnostic [21] and subtype criteria [22] and healthy subjects (n = 11)). Skin biopsies were obtained from over the dorsal mid forearm and immediately stored in RNAlater (Qiagen) at -80°C until RNA isolation. The modified Rodnan skin score (MRSS) was determined for each patient on the day of the biopsy [23].

### RNA isolation and quantitative real-time PCR

Human skin biopsies were placed in RLT lysis buffer (Qiagen), minced and disrupted using a Polytron homogenizer (Capitol Scientific, Austin, TX, USA). RNAs were isolated in accordance with the RNeasy Mini Kit protocol (Qiagen). The concentration of total RNA isolated was measured (Nanodrop 1000; Thermo Scientific) and 200 ng of RNA was used to synthesize complementary DNAs (cDNAs) according to the protocol for SuperScript II reverse transcriptase (Invitrogen) using random primers. cDNAs were used as templates for quantitative real-time PCR analysis with gene expression assays (TaqMan; Applied Biosystems), using the following primer pairs: 18S (4319413E); CD14 (Hs02621496_s1); MD2 (Hs01026734_m1); TLR4 (Hs00152939_m1).

### *In vivo* administration of LPS

Mice wild-type (WT) (C57BI/6 WT), TLR4-/- (B10ScN-*Tlr4lps-del*/JthJ), and TLR2-/- (B6.129-*Tlr2tm1Kir*/J) were obtained from Jackson Laboratories; C57BI/6/MyD88-/-mice were obtained from Dr Shizuo Akira [24]. Briefly, mice were anesthetized by intraperitoneal (i.p.) injection of ketamine (100 mg/kg) and xylazine (5 mg/kg). Osmotic pumps (Alzet) designed to deliver lipopolysaccharide (LPS-EB ultrapure Invivogen: Ultra pure lipopolysaccharide from *Escherichia coli* 0111:B4 strain- TLR4 ligand) at 0.5 mg/ml and 0.1 mg for a total dose of 200 μl released over 7 or 28 days, or sterile PBS (Gibco) were sterilely implanted subcutaneously in 6- to 8-week-old mice. The concentration of LPS used in 1-week pumps was 200 μg/ml and in 4-week pumps it was 800 μg/ml. Thus, the rate of release of LPS per hour was the same in both pump experiments (200 ng of LPS per hour). After 1 week or 4 weeks, mice were sacrificed and skin (approximately 1 cm^2^) surrounding the pump outlet was homogenized in Trizol (Invitrogen) for preparation of RNA and in some experiments fixed in formalin for histology and immunohistochemistry. All the procedures were approved by the institutional animal care and used committee at Boston University Medical Campus.

### Anti TGF-β antibody treatment

To block TGF-β gene expression, WT mice were treated with i.p. injections of anti TGF-β antibodies (α-TGF-β1,2,3, 125 μg/per mouse, R&D Systems,) on the same day of LPS pump insertion, and on days 2 and 5 after pump insertion. Control mice were treated with Isotype IgG1 i.p. injection (125 μg/per mouse, R&D Systems). Mice were sacrificed and skin (approximately 1 cm^2^) surrounding the pump outlet was homogenized in Trizol (Invitrogen) for preparation of RNA or fixed in formalin for immunohistochemistry.

### Monocyte-macrophage depletion

To explore the importance of monocytes/macrophages, we used a macrophage-deficient model achieved by diphtheria toxin (DT) treatment of mice selectively expressing the diphtheria toxin receptor in CD11b + cells. Itgam(CD11b)-DTR (B6.FVB-Tg(ITGAM-DTR/EGFP)34Lan/J) mice were obtained from Jackson Laboratories. These transgenic mice have a CD11b promoter that drives the expression of the human DT receptor leading to the depletion of monocytes after receptor ligation. To induce monocyte/macrophage depletion, 25 ng of DT per gram of body weight was given by i.p. injection on the same day of LPS or PBS pump insertion, and a second time at 48 hours. Mice were sacrificed at day 5. ITGAM-DTR control mice received PBS i.p. injections (CD11b-DTr LPS/PBS). Skin (approximately 1 cm^2^) surrounding the pump outlet was homogenized in Trizol (Invitrogen) for preparation of RNA or fixed in formalin or optimal cutting temperature compound (OCT) for immunohistochemistry or immunofluorescence, respectively. Immunofluorescent staining with CD11b and F4/80 (BD Biosciences) of distal skin was used to document monocyte/macrophage depletion (data not shown).

### Immunohistochemistry

Immunohistochemistry was performed using the Vectastain ABC kit (Vector Laboratories) according to the manufacturer’s instructions on formalin-fixed, paraffin-embedded skin tissue sections. Briefly, sections were deparaffinized, rehydrated in acidic antigen-retrieval solution (pH = 6), and blocked with FC Blocker and Background blocker (Innovex) and normal blocking serum for 30 minutes. Sections were stained with hematoxylin and eosin. The sections were then incubated overnight at 4°C with antibodies against CD163, Arginase-1 (ARG-1) and MAC-3 (CD163: Epitomics, Burlingame, CA, USA, dilution 1:200 in blocking buffer; ARG-1: LifeSpan Biosciences, Inc. dilution 1:250; MAC-3: BD Pharmingen™, dilution 1:100) followed by incubation for 30 minutes with a biotinylated secondary antibody solution. The sections were developed by Dako Chromogen System and counterstained with hematoxylin. Isotype control staining was performed for each antibodies used (data not shown).

### Flow cytometry

For analysis of cellular infiltrate, mouse skin was minced and followed by enzymatic digestion with 0.28 U/ml Liberase 3 (Roche) for 20 minutes at 37°C, passed through a 70-μm filter washed in Roswell Park Memorial Institute medium (RPMI) without serum, and counted. Flow cytometry was performed using fluorochrome conjugated monoclonal antibodies to mouse CD11b (BD Biosciences). Macrophages were identified as CD11b^+^SSC^lo^, and granulocytes as CD11b^+^SSC^hi^. Cells were acquired with the LSRII Flow Cytometer (BD Biosciences) and the data were analyzed with FlowJo software (Tree Star).

### Nanostring analysis

Skin from mice treated with PBS and LPS was analyzed using nanostring technology [25]. A set of 50 genes, including inflammatory genes, macrophages markers, TGF-β-regulated genes, and others, were analyzed: 100 ng of RNA per sample was used and gene expression was normalized to the expression of eight housekeeping genes. The analysis was performed using GraphPad Software, Inc, and clustered by Cluster 3.0 software.

### Statistical analysis

Data were analyzed using the Mann-Whitney test. Correlations were calculated using Pearson correlation and graphed showing the linear regression. All analyses were performed using Prism software (GraphPad Software, Inc.). Differences were considered significant at a *P*-value <0.05.

## Results

### Study patients

All patients selected met the criteria for dcSSc according to diagnostic [21] and subtype criteria [22]. The MRSS [22] was used to determine the extent of skin involvement [23]. The mean age for these patients (n = 24) was 48 ± 11 years (mean ± standard error of the mean (SEM)) with 68% (n = 17) of those studied being female. The average MRSS was 23 ± 11. Of the patients studied, 45% (10 out of 22 patients) were receiving treatment at the time of the biopsy. The mean age for the healthy controls was 40 ± 16 years, and 45% were female.

### TLR4-complex expression in skin from patients with dcSSc

**Figure 1:**
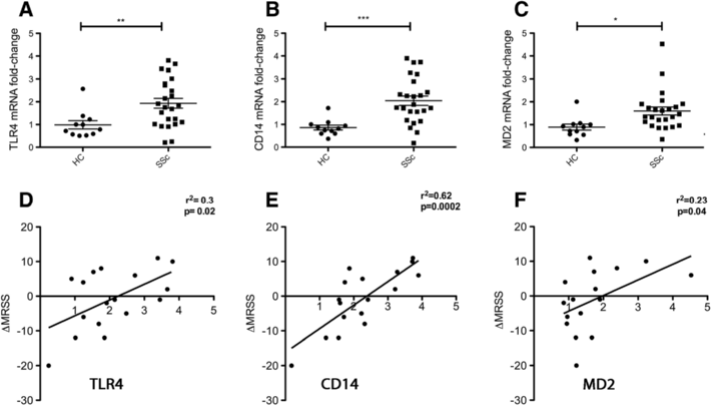
**Overexpression of Toll-like receptor 4 (TLR4)-complex genes in skin from diffuse cutaneous systemic sclerosis (dcSSc) patients. (A-C)** Gene expression comparing dcSSc patients (SSc, n = 24, solid squares) to healthy controls (HC, n = 11, solid circles). Data are expressed as fold-change, normalized to mRNA expression by q-PCR, all expression normalized to one HC: TLR4 **(A)**, CD14 **(B)** and Myeloid differentiation factor 2 (MD2) **(C)** show average SSc mRNA expression increased, respectively, by 2-fold (*P* <0.01); 2.4-fold (*P* <0.0001), and 1.8-fold (*P* <0.05). Bars show the mean ± standard error of the mean. In the dot plot, each data point represents a single skin sample. **(D-F)** Correlation between gene expression and change in skin score; change in skin score was calculated as the difference in the modified Rodnan skin score over 6 months (ΔMRSS). Correlation of ΔMRSS with: **(D)** TLR4 gene expression (*r*^2^ = 0.3, *P* = 0.02); **(E)** CD14 gene expression (*r*^2^ = 0.62, *P* = 0.0002); and **(F)** MD2 gene expression (*r*^2^ = 0.23, *P* = 0.04).

In order to explore TLR4 and its co-receptors in the skin of dcSSc patients, we analyzed the mRNA level of TLR4, CD14, and MD2. Lesional skin from dcSSc patients showed significantly higher levels of TLR4 mRNA compared to skin samples from healthy controls. (Figure 1A; TLR4, 2-fold increase, *P* <0.01). DcSSc skin samples also showed significantly higher expression of the co-receptors CD14 and MD2 (Figure 1B,C CD14, 2.4-fold increase, *P* <0.0001; MD2, 1.8-fold increase, *P* <0.05) compared to skin from healthy controls.

To better understand whether TLR4 and co-receptor expression might contribute to pathogenesis, we examined the relationship between the expression of TLR4 and its co-receptors to the MRSS, a measure of the degree of skin fibrosis in SSc patients [26]. Unlike several biomarkers we have reported previously [27], the MRSS did not correlate with expression of TLR4, CD14 or MD2 (data not shown). Although the MRSS assesses skin disease at the time it is scored, this single measure does not provide information about the disease trajectory, that is, whether skin fibrosis is progressively involving more skin and/or more severely involving skin already affected by the disease process. Comparing the MRSS at the time of the biopsy to a later point in time can make an assessment of disease activity. This is particularly important because we know that skin disease in dcSSc patients cannot only stabilize, but also regress in a significant fraction of patients [28]. Thus we assessed the relationship between TLR4 and co-receptors with progressive skin disease, using delta-MRSS (ΔMRSS), that is, the change in the MRSS at 6 months after the skin biopsy compared to the baseline MRSS assessed at the time of the skin biopsy. For the patients included in our study, the range of ΔMRSS (calculated for 18 patients) was from -20 to 10. TLR4 and MD-2 expression correlated modestly but significantly with ΔMRSS (TLR4: *r*^2^ = 0.3, *P* = 0.02; MD-2: *r*^2^ = 0.23, *P* = 0.04) (Figure 1D, F). Strikingly, CD14 mRNA expression correlated highly with ΔMRSS (*r*^2^ = 0.62, *P* = 0.0002) (Figure 1E), indicating an important connection between CD14 expression and progressive disease in dcSSc patients.

### Chronic dermal LPS exposure induces inflammation in mouse skin

**Figure 2:**
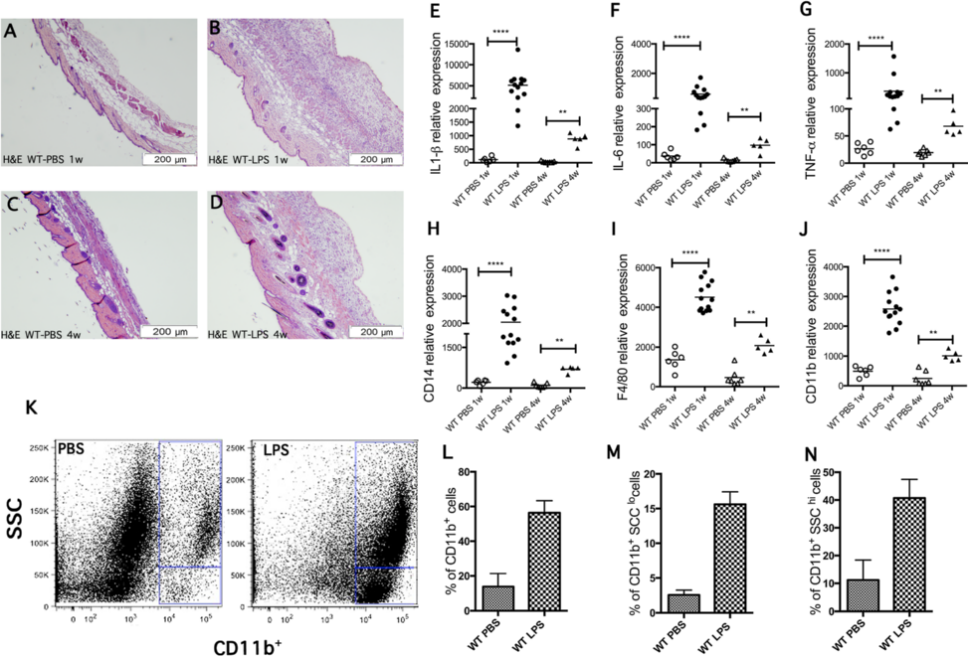
**Toll-like receptor 4 (TLR4) activation induces inflammation and cellular infiltration. (A-D)** Representative figure of H&E-stained cross-section of skin from C57Bl6/wild-type (WT) mouse after treatment with PBS or lipopolysaccharide (LPS) with subcutaneous pumps for 1 week (WT-PBS-1w or WT-LPS-1w: **A**, **B**) or 4 weeks (WT-PBS-4w or WT-LPS-4w: **C**, **D**). **(E-L)** Relative expression of mRNA by nanostring in 1-week, LPS-treated (n = 13, solid circles) compared to PBS-treated (n = 6, open circles) WT mice, and 4-week, LPS-treated (n = 5, solid triangles) compared to PBS-treated (n = 7, open triangles) WT mice, **P <0.01, ****P <0.0001. In the dot plot, each data point represents a single sample**.** Increased gene expression of proinflammatory markers in LPS- compared to PBS-treated mice at 1 week and 4 weeks: **(E)** IL-1β: 1 week, *P* <0.0001; 4 weeks, *P* <0.01. **(F)** IL-6: 1 week, *P* <0.001; 4 weeks, *P* <0.01. **(G)** TNF-α: 1 week, *P* <0.0001; 4 weeks, *P* <0.01. **(H)** CD14: 1 week, *P* <0.0001; 4 weeks, *P* <0.01. **(I)** F4/80: 1 week, *P* <0.0001; 4 weeks, *P* <0.01. **(J)** CD11b: 1 week, *P* <0.0001; 4 weeks, *P* <0.001. **(K)** Flow cytometry: representative plots of CD11b^+^ high-scatter (CD11b^+^SSC^hi^) and low-scatter (CD11b^+^SSC^lo^) cells, isolated from skin of WT mice treated with PBS or LPS for 1 week. **(L-N)** Percentage of cells, CD11b^+^**(L)**, CD11b^+^SSC^hi^**(M)**, and CD11b^+^SSC^lo^**(N)** cells in LPS-treated WT mice (n = 3) compared with PBS-treated WT mice (n = 3), for 1 week. Each bar represents the mean ± standard error of the mean.

To better understand the effect in skin of TLR4 activation *in vivo*, we tested the effect of continuous stimulation with LPS for 1 or 4 weeks by subcutaneous osmotic pump. Skin histology from the infused site of mice treated with LPS for 1 or 4 weeks showed similar striking inflammation in the subcutaneous, deep dermis, and fat layers (Figure 2A-D).

To further investigate the effect of LPS in skin, we analyzed expression of proinflammatory genes: IL-1β, IL-6, and TNF-α. All three of these inflammatory cytokines were strikingly elevated in mice treated with LPS for 1 week compared with controls, PBS treated mice (IL-1β, 43.79-fold *P* <0.0001; IL-6, 15.74-fold; *P* <0.0001; and TNF-α, 13.31-fold increase, *P* <0.0001, Figure 2E-G). Skin from mice treated for 4 weeks with LPS showed a less striking increase in IL-1β, IL-6, and TNF-α (IL-1β, 25.24-fold, *P* <0.01; IL-6, 6.81-fold, *P* <0.01; TNF-α, 3.50-fold increase, *P* <0.01; Figure 2E-G).

### Chronic dermal LPS exposure recruits macrophages and granulocytes

In order to quantitatively define the inflammatory cell types recruited to the skin by LPS treatment, we analyzed the expression of immune cell markers using flow cytometry on cells extracted from treated skin (Figure 2K-N). After 1 week of LPS treatment we found a remarkable increase of CD11b-positive cells (56%, mean of three experiments of CD11b^+^ cells) compared to PBS-treated skin (13%, mean of three experiments of CD11b^+^ cells) (WT PBS compared to WT LPS: 4.1-fold increase in the percentage of cells; Figure 2L). This population could be further divided based on the expression of CD11b and side scatter (SSC), a measure of cell granularity or internal complexity. We found two groups of cells: CD11b^+^SSC^lo^ (identifying macrophages), and CD11b^+^SSC^hi^ (identifying granulocytes). Both of these populations were increased in LPS-treated skin compared to PBS-treated control skin (CD11b^+^SSC^hi^, 3.6-fold increase in percentage of cells; CD11b^+^SSC^lo^, 6.1-fold increase in percentage of cells, Figure 2M-N). The highly induced infiltration of these two cell types is consistent with histologic evaluations noted above (see Figure 2A-D). In contrast, the number of T cells and B cells (marked with CD3 and B220, respectively) did not show any difference between mice treated with LPS compared to PBS-treated skin (data not shown).

LPS-treated skin showed increased mRNA expression of macrophage markers, including CD14 (9.54-fold increase, *P* <0.0001), F4/80 (3.32-fold increase, *P* <0.0001), and CD11b (5.27-fold increase, *P* <0.0001) after 1 week of LPS (Figure 2H-J). We also observed significant changes in mRNA levels of these genes at 4 weeks (CD14, 6.78-fold increase, *P* <0.01; F4/80, 4.52-fold increase, *P* <0.01; CD11b, 4.12-fold increase, *P* <0.01; Figure 2H-J). In contrast to these monocyte/macrophage markers and consistent with our flow cytometry analyses, mRNA levels of T-cell (CD3) and B-cell (CD19) markers showed no change in LPS-treated compared to PBS-treated control skin at 1 or 4 weeks (data not shown).

### Dermal LPS exposure activates M1 and M2 macrophages

**Figure 3:**
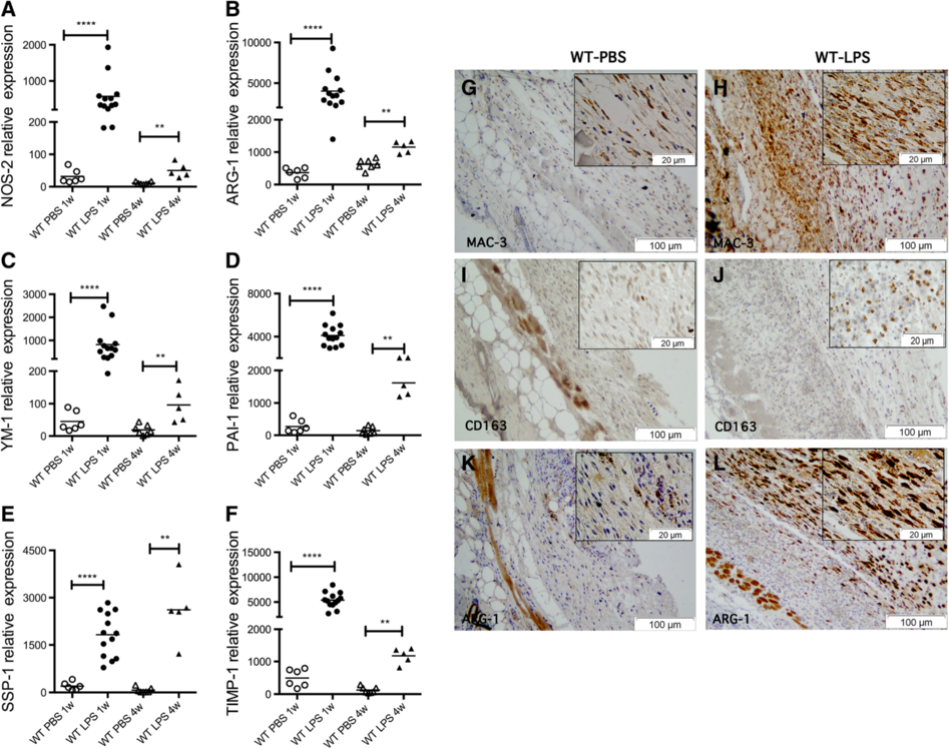
**Lipopolysaccharide (LPS) induces overexpression of M1 and M2 macrophage markers, and transforming growth factor (TGF)-β signature genes. (A-F)** Gene expression by nanostring, comparing 1-week, LPS- (n = 13, solid circles) with PBS- (n = 6, open circles) treated wild-type (WT) mice; and 4-week LPS- (n = 5, solid triangles) with PBS-treated (n = 7, open triangles) WT mice. **P <0.01, ****P <0.0001. In the dot plot, each data point represents a single sample**.** Increased gene expression in LPS- compared to PBS-treated WT mice of macrophage markers: **(A)** NOS2, 1 week, *P* <0.0001; 4 weeks, *P* < 0.001. **(B)** Arginase-1 (ARG-1), 1 week, *P* <0.0001; 4 weeks, *P* <0.01. **(C)** YM1, 1 week, *P* <0.0001; 4 weeks, *P* <0.01. **(D)** Plasminogen activator inhibitor-1 (PAI-1), 1 week, *P* <0.0001; 4 weeks, *P* <0.01. **(E)** SPP1, 1 week, *P* < 0.0001; 4 weeks, *P* <0.01. **(F)** TIMP1, 1 week, *P* <0.0001; 4 weeks, *P* <0.01. **(G-L)** Representative images of MAC-3 **(G, H)**, CD163 **(I, J)**, and ARG-1 **(K, L)** staining on skin section from WT mice treated with PBS (WT-PBS: **G**, **I**, **K**) or LPS (WT-LPS: **H**, **J**, **L**) for 1 week.

To characterize the M1 and M2 phenotype of macrophages recruited by LPS, we analyzed expression of mRNA levels of the M1 macrophage marker NOS2 (Figure 3A), and M2 macrophage markers, ARG-1 and YM1 (Figure 3B, C). Although TLR4 activation *in vitro* is typically associated with M1 macrophages, both the M1 marker, NOS2, and the M2 markers, ARG-1 and YM1, were strikingly and significantly increased in mice treated with LPS for 1 week (NOS2, 18.03-fold increase, *P* <0.0001; ARG-1, 10.98-fold increase, *P* <0.0001; YM1, 18.20-fold increase, *P* <00001). After 4 weeks of LPS treatment, we observed a less striking increase of the M1 and M2 macrophage markers (NOS2, 4.65-fold increase, *P* <0.01; ARG-1, 1.85-fold increase, *P* <0.01; YM1, 5.08-fold increase, *P* <0.01; Figure 3A-C).

Immunostaining on paraffin sections of Mac-3, a general marker for macrophages (Figure 3G, H), and CD163 (Figure 3I, J) and ARG-1 (Figure 3K, L), M2 markers supported above observations. Highly increased numbers of CD163- and ARG-1-positive cells stained primarily in the subcutaneous region of mice treated with LPS for 1 week compared with PBS-treated controls. Mac-3-positive cells were well-distributed in all skin layers, mostly in the LPS-treated mice compared to the PBS controls. After 4 weeks, we observed more Mac3-, CD163- and Arg-1-positive cells in the skin of mice treated with LPS compared with controls, but the staining was much less intense compared to staining in 1-week LPS-treated mice, consistent with the lower level of M2 gene expression (Additional file 1).

### LPS treatment induces pro-fibrotic gene expression and fibrosis

Recent studies have suggested that TLR4 activation is involved in the amplification of fibrosis and TGF-β responses in SSc, and TLR4 activation has been implicated in other fibrotic diseases [17-19]. To investigate the potential role of TLR4 activation *in vivo*, we monitored the expression of several TGF-β regulated genes in our murine model. We have recently identified several genes regulated by TGF-β in mouse skin [3], that are also increased in the skin of dcSSc patients, including PAI-1 (Serpine-1) [27], SPP1 (osteopontin 1), and TIMP1 (tissue inhibitor metalloproteinase 1, unpublished data). After 1 week of LPS treatment, we observed significantly increased expression of PAI-1 (15.01-fold increase, *P* <0.0001; Figure 3D), SPP1 (9.14-fold increase, *P* <0.0001; Figure 3E), and TIMP1 (10.77-fold increase, *P* <0.0001; Figure 3F), in LPS-treated mice (Figure 3D-F). Additionally, we found increased expression of other TGF-β regulated genes: COL5A1 (Collagen type 5, alpha 1), LOX (lysyl oxidase), SFRP2 (Secreted frizzled-related protein 2), MMP13 (matrix metallopeptidase 13), MMP3 (matrix metallopeptidase 3), WISP1 (WNT1-inducible-signaling pathway protein 1), and THBS1 (thrombospondin 1) in the mice treated with LPS after 1 and 4 weeks compared to the controls (Additional file 2). We observed similar increases in TGF-β-regulated genes in LPS-treated skin at 4 weeks (PAI-1, 11.22-fold increase, *P* <0.01; SPP1, 9.14-fold increase, *P* <0.01; TIMP1, 9.24-fold increase, *P* <0.01; Figure 3D-F).

### Inhibition of TGF-β signature gene expression in LPS-treated mice by anti-TGF-β antibody (α-TGF-β) treatment and upon macrophage depletion

**Figure 4:**
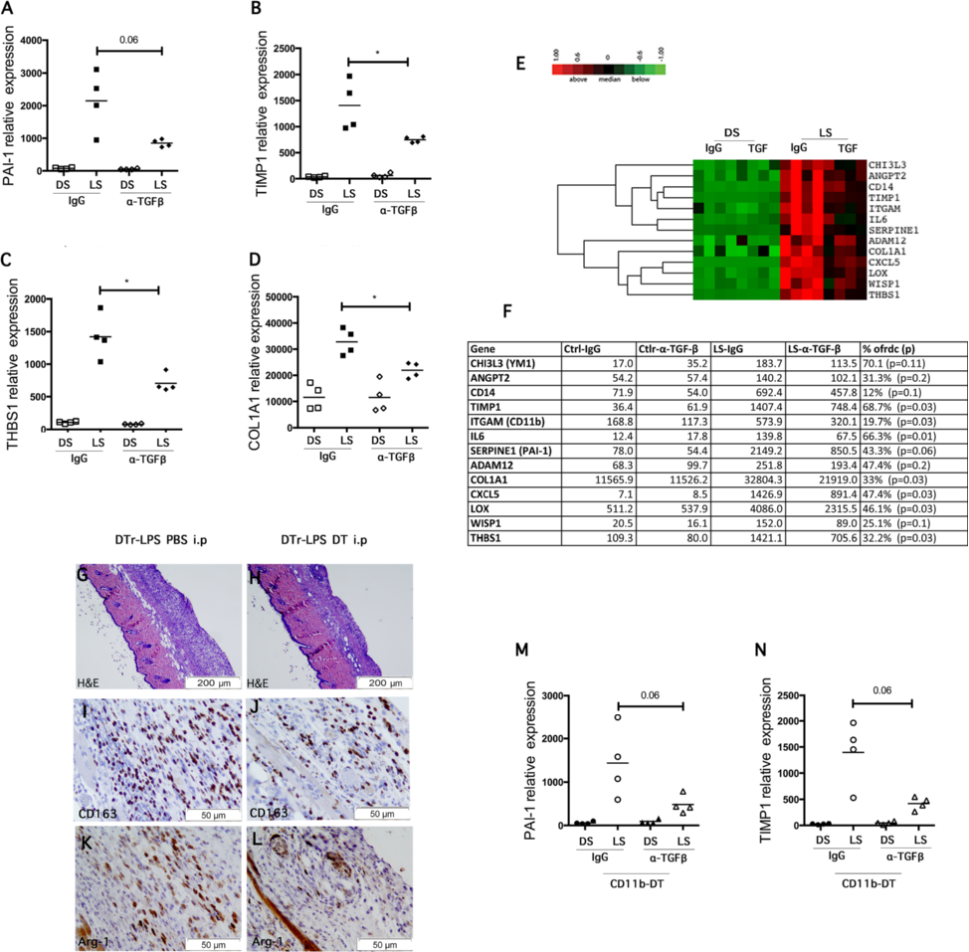
Transforming growth factor (TGF)-β signature gene is reduced after anti-TGF-β antibody (α-TGF-β) injection and after macrophage depletion

As upregulated TGF-β signature genes were observed in LPS-treated mouse skin, we tested whether α-TGF-β would block expression of these genes. Mice treated with α-TGF-β showed a remarkable reduction in the expression of PAI-1 (LPS-treated, WT-IgG compared to WTα-TGF-β, 2.53-fold decrease, *P* = 0.06), TIMP1 (LPS-treated, WT-IgG compared to WT α-TGF-β, 1.88-fold decrease, *P* <0.05), THBS1 (LPS-treated, WT-IgG compared to WTα-TGF-β, 2.01-fold decrease, *P* <0.05) and COLA1A (LPS-treated, WT-IgG compared to WTα-TGF-β, 1.5-fold decrease, *P* <0.05) compared to control mice treated with isotype-matched Ig (Figure 4A-D). Supervised clustering of the cutaneous gene expression of these mice, showed other genes that were significantly reduced after α-TGF-β treatment, such as CXCL5, CD14, CD11b and IL-6, suggesting that the expression of these genes is induced by TGF-β in our model (Figure 4E, F).

To further investigate the pro-fibrotic mechanism induced by LPS and related to the activation of M2 macrophages, we tested chronic LPS exposure in a macrophage-deficient model. Transgenic mice selectively expressing the diphtheria toxin receptor (DTr) in CD11b^+^ cells were treated with DT or PBS (control) and LPS pumps inserted subcutaneously. Mice injected with DT (Figure 4G, CD11b-DTr LPS/DT) did not show the striking cellular infiltration (Figure 4H, CD11b-DTr LPS/DT) or the overexpression of inflammatory genes (data not shown) seen in control mice. Despite only partial depletion of macrophages in the skin treated with LPS, as shown by the presence of some CD163- and ARG-1-positive cells (Figure 4I-L), the expression of most of the pro-fibrotic genes were significantly decreased compared to the controls, PAI-1 (LPS-treated mice, CD11b-DTr LPS/PBS compared to CD11b-DTr LPS/DT, 2.53-fold decrease, not significant, *P* = 0.06; Figure 4K) and PBS compared to CD11b-DTr LPS/DT, 3.32-fold decrease, *P* = 0.06; Figure 4L). Other inflammatory and pro-fibrotic genes were also significantly blocked such as, IL-1 β, IL-6, COLA1A, WISP and MMP12 (data not shown).

### Inhibited proinflammatory and pro-fibrotic effects of LPS in TLR4- and MyD88- deficient mice

**Figure 5:**
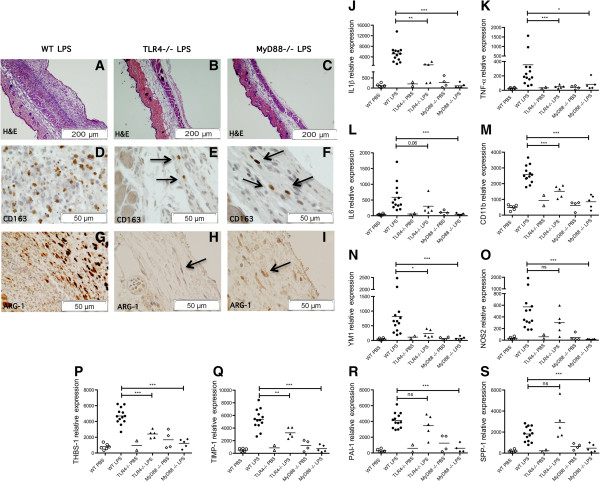
**Effects of lipopolysaccharide (LPS) treatment are partially inhibited in Toll-like receptor 4 (TLR4)-deficient mice and blocked in Myeloid differentiation factor 88 (MyD88)-deficient mice. (A-F)** Representative of H&E **(A-C)**, CD163 **(D**-**F)**, and arginase-1 (ARG-1) **(G-I)** staining cross section of murine skin after LPS-pump in wild-type (WT) **(A, D, G)**, TLR4-/- **(B, E, H)** or MyD88-/- **(C, F, I)** mice. **(J-R)** Gene expression in skin of WT, TLR4-/- and MyD88-/- mice treated for 1 week with PBS: WT (n = 6, open circles) TLR4-/- (n = 2, open triangles), MyD88-/- (n = 4, open diamonds); or with LPS: WT (n = 13, solid circles) TLR4-/- (n = 5, solid triangles), MyD88-/- (n = 6, solid diamonds); *P <0.05, ** *P* <0.01, *** *P* <0.001, ns = not significant. Proinflammatory genes: **(J)** IL-1β, **(K)** TNF-α, and **(L)** IL-6. Macrophage markers: **(M)** CD11b; M2 and M1 macrophages: **(N)** YM1 and **(O)** NOS2. Pro-fibrotic genes: **(P)** thrombospondin 1 (THBS1), **(Q)** tissue inhibitor metalloproteinase 1 (TIMP1), **(R)** plasminogen activator inhibitor-1 (PAI-1) and **(S)** osteopontin 1 (SPP1).

To define the contribution of TLR4 activation and the consequent recruitment and activation of the adaptor molecule MyD88, to inflammation and fibrosis, we tested LPS treatment in TLR4- and MyD88-deficient mice for 1 week by subcutaneous osmotic pump. H&E staining from LPS-treated, TLR4- and MyD88-deficient mice showed an important reduction of cellular infiltration in the subcutaneous layer (Figure 5B, C), compared to the WT LPS-treated mice (Figure 5A). Furthermore, we observed that the expression of inflammatory cytokines, TNF-α and IL-6, were almost completely blocked in skin of LPS-treated, TLR4- and MyD88- deficient mice compared to WT mice (TNF-α, LPS-treated WT compared to TLR4-/-, 7-fold decrease, *P* <0.001; LPS-treated, WT compared to MyD88-/-, 50.49-fold decrease, *P* <0.05; IL-6, LPS-treated, WT compared to TLR4-/-, 1.93-fold decrease, not significant, *P* = 0.6; LPS-treated, WT compared to MyD88-/-, 304.37-fold decrease, *P* <0.001; Figure 5K, L). In contrast, IL1β was blocked in LPS-treated MyD88-deficient mice, but showed only a trend toward partial inhibition in LPS-treated TLR4-deficient mice compared to controls (IL1β, LPS-treated, WT compared to TLR4-/-, 5.7-fold decrease, *P* <0.01; LPS-treated, WT compared to MyD88-/-, 1014.82-fold decrease, *P* <0.001; Figure 5J).

To further investigate signals mediating macrophage activation, we analyzed M1 and M2 macrophage markers in TLR4- and MyD88-deficient mice. LPS-treated, TLR4- and MyD88-deficient mice showed few CD163- and ARG-1-positive cells compared to LPS-treated WT mice (Figure 5D-I). mRNA levels of CD11b (Figure 5M), and the M2 marker, YM1 (Figure 5N), were significantly reduced in LPS-treated, TLR4- and MyD88-deficient mice compared to LPS-treated WT mice (LPS-treated, WT compared to TLR4-/-, CD11b, 1.70-fold decrease, *P* <0.001; YM1, 3.48-fold decrease, *P* <0.05; LPS-treated, WT compared to MyD88-/-, CD11b, 1516.06-fold decrease, *P* <0.001; YM1, 172.64-fold decrease, *P* <0.001; Figure 5M, N). NOS2 expression was completely blocked in LPS-treated MyD88-deficient mice, but not in LPS-treated TLR4-deficient mice (LPS-treated, WT compared to TLR4-/-, 1.90-fold decrease, not significant, *P* = 0.22; LPS-treated, WT compared to MyD88-/-, 218.34-fold decrease, *P* <0.001; Figure 5O).

Notably, induction of TGF-β genes was completely blocked in LPS-treated, MyD88- deficient mice (THBS1, WT compared to MyD88-/-, 2,409.04-fold decrease, *P* <0.001; TIMP1, LPS-treated, WT compared to MyD88-/-, 3,249.62-fold decrease, *P* <0.001; PAI -1, LPS-treated, WT compared to MyD88-/-, 3,466.56-fold decrease, *P* <0.001; SPP-1, LPS-treated, WT compared to MyD88-/-, 2,908.35-fold decrease, *P* <0.001; Figure 5P-S). THSB1 and TIMP1 were also significantly decreased in LPS-treated TLR4-deficient mice but slightly increased compared to the controls (THBS1, LPS-treated, WT compared to TLR4-/-, 1.95-fold decrease, *P* <0.001; TIMP1, LPS-treated, WT compared to TLR4-/-, 1.66-fold decrease, *P* <0.01; Figure 5P, Q). On the other hand, in LPS-treated, WT and TLR4-deficient mice, mRNA levels of PAI-1 and SPP-1 were similar (PAI-1, LPS-treated, WT compared to TLR4-/-, 1.18-fold decrease, not significant, *P* = 0.36; SPP-1, LPS-treated, WT compared to TLR4-/-, 0.63-fold increase, not significant, *P* = 0.20; Figure 5R, S). As TLR4-deficient mice still responded partially to LPS, we also tested LPS in TLR2-deficient mice. However, TLR2-deficient mice showed no difference in gene expression compared to WT, LPS-treated mice (Additional file 3).

## Discussion

We show here that TLR4 co-receptors, CD14 and MD2, are overexpressed in the skin of dcSSc patients. As CD14 is expressed mainly on macrophages and monocytes, these current findings are in line with previous studies published by our group, showing that the macrophage markers, Siglec1 (sialic acid-binding immunoglobulin-type lectin-1) and MRC1 (mannose receptor-1) are increased in lesional skin in SSc [3,29], suggesting an important function of innate immunity and particularly of macrophages in the tissue inflammation and fibrosis of skin in SSc. Expression of MD2, TLR4 and, most strikingly, CD14 correlated with progressive skin disease, as assessed by the change in MRSS 6 months after the skin biopsy was performed. Thus, expression of these genes and in particular CD14 appears to be the first prognostic biomarker identified in SSc skin. Prognostic biomarkers are particularly important in this disease because of the extremely variable course of skin disease, progressing in some patients while regressing in others, and because clinical markers of disease provide limited information about disease trajectory [30]. CD14 expression may provide important information about the likelihood of progression and thus the need for treatment. As most of the patients in this study were taking some type of immunosuppressive medication, these correlations may be confounded by the treatment regimen, the most likely effect to decrease the apparent degree of correlation, because patients responding to a treatment might deviate from the anticipated natural history. Therefore, these correlations may underestimate the prognostic utility of CD14 expression, however, further studies will be required to clarify how strongly CD14 expression in the skin correlates with progressive skin disease.

Chronic skin exposure to endotoxin (LPS) induced inflammation, cellular recruitment and activation of TGF-β signature genes. It is well known that LPS is a powerful immunostimulant, and indeed, LPS-treated mice, at both 1 and 4 weeks, showed inflammation, characterized by macrophage infiltration and induction of proinflammatory chemokines. However, chronic stimulation of TLR4 at a low concentration of LPS can induce endotoxin tolerance characterized by a transient unresponsive state, reduced proinflammatory response, cellular desensitization, and cellular reprogramming [31]. This has been attributed to induction by LPS of a variety of immune modulators, including IRAK-M, SOCS-1, and IL-10 [32]. In our study, we found that the levels of the proinflammatory chemokines in 4-week LPS-treated mice were lower than in 1-week LPS-treated mice, suggesting that longer activation of TLR4 might induce endotoxin tolerance, activating counter-regulatory processes that limit inflammation.

Pathways activated by endotoxin tolerance might explain the presence of M2 macrophages found most strongly at 1 week in LPS-treated mice: 1-week LPS-treated mice showed severe macrophage and granulocyte infiltration, the first cells recruited in the early phase of inflammation. These findings are consistent with the markedly increased staining of Mac-3, a general marker for macrophages, and Arg-1 and CD163, M2 macrophage markers, in 1-week LPS treated skin. These data suggest that LPS stimulation activates both M1 and M2 macrophages, and support *in vitro* studies performed on human macrophages, showing that endotoxin tolerance induces M2 as well as M1 macrophages. M2 macrophages may be important in the pro-fibrotic response either by directly releasing pro-fibrotic cytokines or by recruiting other cell types that regulate extracellular matrix turnover [33,34].

TGF-β signature genes, PAI-1 and TIMP1, showed highly increased expression at both 1- and 4-week time points, showing that chronic exposure to endotoxin actives pro-fibrotic gene expression. We have recently shown that PAI-1 and SPP1 are overexpressed in the skin of mice treated with TGF-β [3]. Collagen deposition was located mostly in the fascia, rather than the dermis, indicating that LPS and the associated inflammatory response is localized to the site of ligand release. Other *in vivo* studies support several *in vitro* studies, showing that TLR4 activation regulates the TGF-β pathway [20]. Furthermore after LPS exposure the reduction of TGF-β regulated genes, following the injection of anti-TGF-β or macrophage depletion, indicates the involvement of TLRs and mostly MyD88 pathway activation in the induction of a TGF-β signature in the skin. The reduction of pro-fibrotic gene expression after macrophage depletion shows the significant involvement of the macrophages in the production or activation of TGF-β.

To examine the receptor utilized by LPS in our model, we compared the effect of LPS treatment in TLR4- and MyD88-deficient mice. Surprisingly, TLR4-deficient mice still responded partially to LPS, showing nearly the same increases in IL-1β, but largely blocked IL-6, TNF-α responses, and cellular infiltration of M2 macrophage markers. LPS also induced some TGF-β responsive genes (PAI-1 and SPP1) in TLR4-deficient mice, whereas deletion of MyD88 blocked all TGF-β regulated gene expression. Although LPS responses seem to generally depend on TLR4 expression [35], LPS has been described to activate other receptors, mainly expressed on macrophages (β-2 integrins, CD11/CD18, moesin, decay accelerating factor (DAF) and CD55) [36-38] or can be spontaneously internalized [38,39]. Our data might suggest that in TLR4-deficient mice LPS actives one of these or other alternative receptors on resident skin cells, macrophages, fibroblasts, keratinocytes, dendritic or endothelial cells. In our model, TLR4 seems to be necessary for macrophage activation. These non-TLR4-mediated effects do not appear to depend on TLR2, as expression in TLR2-deleted mice was similar to WT mice. The complete inhibition in MyD88-deleted mice strongly supports the importance of this pathway in TLR4-mediated fibrosis. Taken together, our findings suggest that the pro-fibrotic and inflammatory signature of our chronic LPS skin model is dependent on MyD88 and might suggest its importance in SSc pathogenesis.

## Conclusion

In conclusion, we showed that TLR4 co-receptors, CD14 and MD2, are overexpressed in the skin of dcSSc patients. Expression of these genes, and in particular CD14, appears to be the first prognostic biomarker identified in SSc skin. In the murine skin, chronic exposure to LPS induced inflammation, cellular recruitment and activation of TGF-β signature genes. We delineated the role of MyD88 as necessary for the induction not only for the early phase of inflammation, but also for pro-fibrotic gene expression via activation of macrophages. Chronic LPS exposure might be a model for the early stage of SSc when inflammation and macrophage activation are important pathological features of the disease. This supports a role for innate immune activation in SSc skin fibrosis, suggesting its importance in SSc pathogenesis.

## Abbreviations

ARG-1: arginase-1; BB20 NOS2: nitric oxide synthase; CD11b: cluster of differentiation molecule 11b; CD14: cluster of differentiation molecule 14; CD163: cluster of differentiation molecule 163; CD18: cluster of differentiation molecule 18; CD3: cluster of differentiation molecule 3; CD55: cluster of differentiation molecule 55; cDNA: complementary DNA; COL1A1: collagen type 1, alpha 1; COL5A1: collagen type 5, alpha 1; CTGF: connective tissue growth factor; CXCL5: C-X-C motif chemokine 5; DAF: decay accelerating factor; DAMPs: damage associated molecular patterns; dcSSc: diffuse cutaneous SSc; DT: diphtheria toxin; DTR: diphtheria toxin receptor; ECM: extracellular matrix; Fn-EDA: fibronectin extradomein A; H&E: hematoxylin and eosin; HMGB1: high mobility group box 1 protein; Hsp: heat-shock proteins; IL: interleukin; Itgam: integrin alpha M; LBP: lipopolysaccharide-binding protein; LOX: lysyl oxidase; LPS: lipopolysaccharide; MD2: myeloid differentiation factor-2; MMP: matrix metallopeptidase; MRSS: modified Rodnan skin score; MyD88: myeloid differentiation factor 88; OCT: optimal cutting temperature compound; PAI-1: plasminogen activator inhibitor-1; PAMPs: pathogen associated molecular patterns; PBS: phosphate buffered saline; qPCR: quantitative real-time polymerase chain reaction; RPMI: Roswell Park Memorial Institute medium; SFRP2: Secreted frizzled-related protein 2; SLE: systemic lupus erythematosus; SPP1: osteopontin 1; SSC: side-scatter; SSc: systemic sclerosis; TGF-β: transforming growth factor-β; THBS1: thrombospondin 1; TICAM: Toll-interleukin 1 molecule-containing adaptor molecule; TIMP1: tissue inhibitor metalloproteinase 1; TIR: Toll-interleukin 1 (IL-1) receptor; TLR: Toll-like receptor; TNF-α: tumor necrosis factor-α; TSLP: thymic stromal lymphopoietin; WISP1: WNT1-inducible-signaling pathway protein 1; WT: wild-type; α-TGF-β: anti-transforming growth factoer-β antibodies; ΔMRSS: delta modified Rodnan skin score.

## Competing interests

The authors declare that they have no competing interests. Supported by the NIH (National Institute of Arthritis and Musculoskeletal and Skin Diseases grant 1P50-AR-060780-02 to Boston University Medical Center and grant 2R01-AR-051089-06A1 to Dr Lafyatis). Mr Affandi’s work was supported by the Dutch Arthritis Association (Reumafonds grant NR-10-1-301) and the Netherlands Organization for Scientific Research (NWO Mosaic grant 017.008.014).

## Authors’ contributions

GS performed all the experimental work and data analysis, the conception and design, and drafted the manuscript. AJA performed the flow cytometry analysis. AJA and AM contributed to the sample collection and the analysis of the data. SN and RBC contributed to the analysis of the data and drafted the manuscript. BN, LMR and JL contributed towards sample collection and preparation. RL designed the study, reviewed the data and prepared the manuscript. All authors read and gave final approval of the version to be published.

## Supplementary Material

Additional file 1 **M1 and M2 markers in 4-week treated mice.** Representative images of Mac-3, **(A, B)**, CD163 **(C, D)**, and arginase-1 (ARG-1) (**E**, **F**) staining on skin section from wild-type (WT) mice treated with PBS (WT-PBS: G, I, K) or LPS (WT-LPS:H, J, L) for 4 weeks. Click here for file

Additional file 2 **Pro-fibrotic gene expression in lipopolysaccharide (LPS)-treated skin.** Pro-fibrotic gene expression in 1- and 4-week LPS-treated mice. In the second and third columns, it is reported as the fold-increase for each pro-fibrotic gene described in the first column, respectively in mice and in 4-week LPS-treated mice (Fold inc. 1-week and Fold inc. 4-week). In the last 2 columns, the *P*-values for each pro-fibrotic gene are reported (*P*-value 1-week and *P*-value 4-week). Fold-increase is calculated as the ratio of the mean of wild-type (WT) LPS-treated and WT PBS-treated mice gene expression. Click here for file

Additional file 3 **Toll-like receptor 2 (TLR2) deficiency does not block lipopolysaccharide (LPS)-induced gene expression.** Gene expression by nanostring, comparing 1-week LPS-treated mice, Distal skin (DS) with local skin (LS) from wild-type (WT) (open circles, DS (n = 2); closed circles, LS (n = 13)), TLR4-/- (open triangles, DS (n = 2); closed triangles, LS (n = 5)) and TLR2-/- (open diamonds, DS (n = 2); closed diamonds, LS (n = 3)) mice, *P <0.05; **P <0.01; ***P <0.001; ****P <0.0001; ns, not significant. In the dot plot, each data point represents a single sample, and the axis a log scale. (**A**) IL-1β (LPS-treated, TLR4-/- compared to WT: 5.07-fold decrease, *P* <0.01; LPS-treated, TLR2-/- compared to WT: 1.25-fold decrease, ns *P* = 0.71); (**B**) THBS1 **(**LPS-treated, TLR4-/- compared to WT: 1.95-fold decrease, ***P <0.001; LPS-treated, TLR2-/- compared to WT: 1.32-fold decrease, ns, *P* = 0.20); (**C**) **PAI-1** (LPS-treated, TLR4-/- compared to WT: 1.18-fold decrease, ns, *P* = 0.36; LPS-treated, TLR2-/- compared to WT: 0.75-fold decrease, ns, *P* = 0.24) and macrophages markers (**D**) CD11b (LPS-treated, TLR4-/- compared to WT: 1.70-fold decrease, ***P <0.001; LPS-treated, TLR2-/- compared to WT: 0.9-fold decrease, ns, *P* = 0.61); (**E**) NOS2 (LPS-treated, TLR4-/- compared to WT: 1.90-fold decrease, ns, *P* = 0.29; LPS-treated, TLR2-/- compared to WT: 1.23-fold decrease, ns, *P* = 0.90; and (F) YM-1 (LPS-treated, TLR4-/- compared to WT: 3.44-fold decrease, ns, *P* = 0.09; LPS-treated, TLR2-/- compared to WT: 1.17-fold decrease, ns, *P* = 0.63). Click here for file
