# Supplementary figures and images for: Chronic Toll-like receptor 4 stimulation in skin induces inflammation, macrophage activation, transforming growth factor beta signature gene expression, and fibrosis
Source: Arthritis Res Ther. 2014 Jul 1;16(4):R136. doi: 10.1186/ar4598 (PMC4227089; doi:10.1186/ar4598)

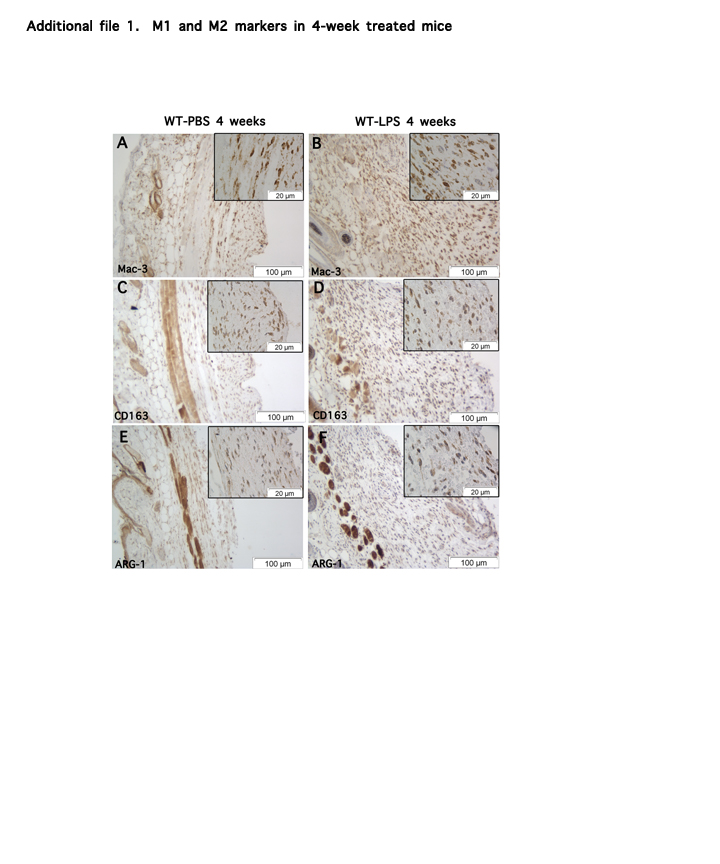

Supplement: Additional file 1 — M1 and M2 markers in 4-week treated mice. Representative images of Mac-3, (A, B), CD163 (C, D), and arginase-1 (ARG-1) (E, F) staining on skin section from wild-type (WT) mice treated with PBS (WT-PBS: G, I, K) or LPS (WT-LPS:H, J, L) for 4 weeks. [file ar4598-S1.jpeg]

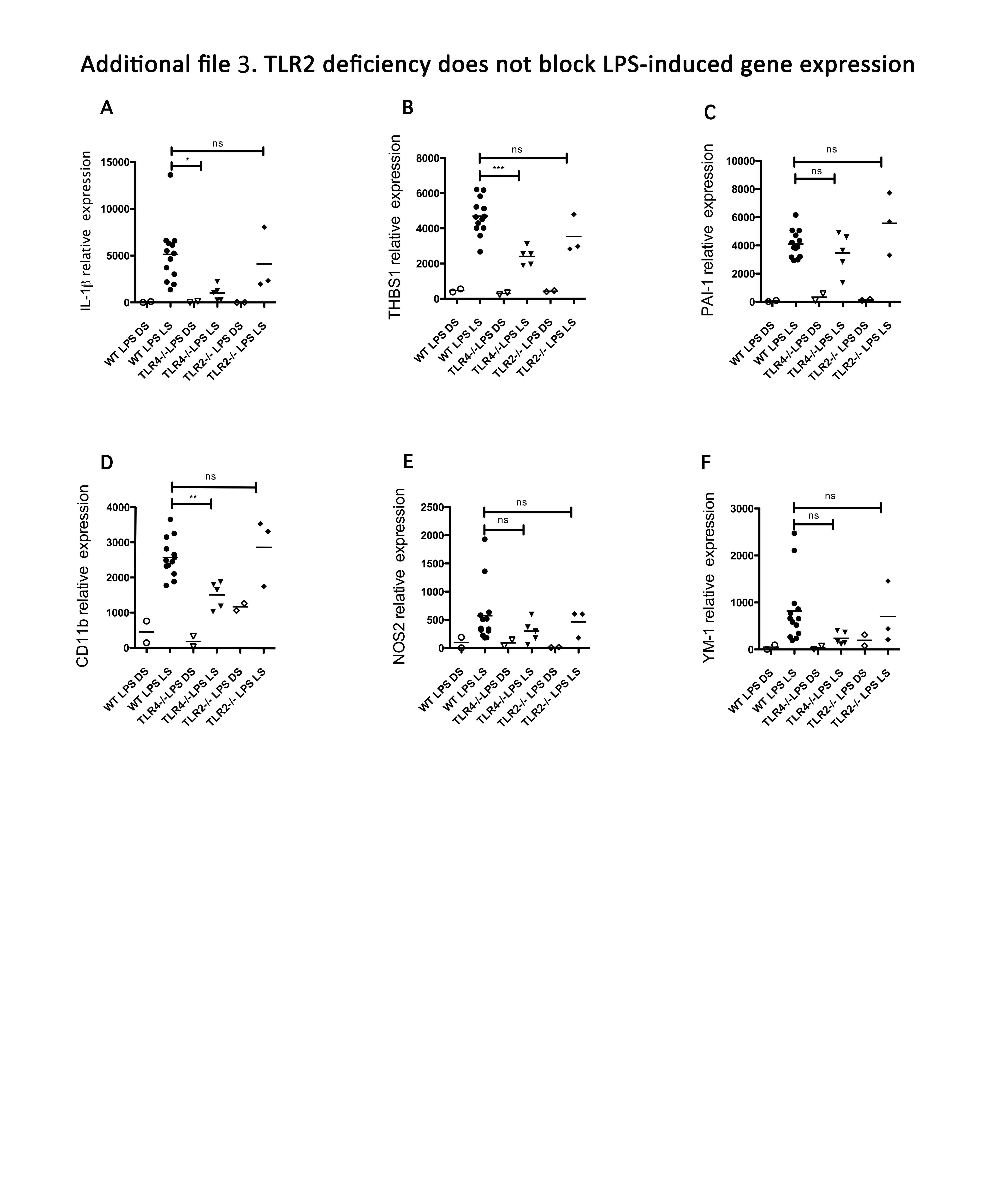

Supplement: Additional file 3 — Toll-like receptor 2 (TLR2) deficiency does not block lipopolysaccharide (LPS)-induced gene expression. Gene expression by nanostring, comparing 1-week LPS-treated mice, Distal skin (DS) with local skin (LS) from wild-type (WT) (open circles, DS (n = 2); closed circles, LS (n = 13)), TLR4-/- (open triangles, DS (n = 2); closed triangles, LS (n = 5)) and TLR2-/- (open diamonds, DS (n = 2); closed diamonds, LS (n = 3)) mice, *P <0.05; **P <0.01; ***P <0.001; ****P <0.0001; ns, not significant. In the dot plot, each data point represents a single sample, and the axis a log scale. (A) IL-1β (LPS-treated, TLR4-/- compared to WT: 5.07-fold decrease, P <0.01; LPS-treated, TLR2-/- compared to WT: 1.25-fold decrease, ns P = 0.71); (B) THBS1 (LPS-treated, TLR4-/- compared to WT: 1.95-fold decrease, ***P <0.001; LPS-treated, TLR2-/- compared to WT: 1.32-fold decrease, ns, P = 0.20); (C) PAI-1 (LPS-treated, TLR4-/- compared to WT: 1.18-fold decrease, ns, P = 0.36; LPS-treated, TLR2-/- compared to WT: 0.75-fold decrease, ns, P = 0.24) and macrophages markers (D) CD11b (LPS-treated, TLR4-/- compared to WT: 1.70-fold decrease, ***P <0.001; LPS-treated, TLR2-/- compared to WT: 0.9-fold decrease, ns, P = 0.61); (E) NOS2 (LPS-treated, TLR4-/- compared to WT: 1.90-fold decrease, ns, P = 0.29; LPS-treated, TLR2-/- compared to WT: 1.23-fold decrease, ns, P = 0.90; and (F) YM-1 (LPS-treated, TLR4-/- compared to WT: 3.44-fold decrease, ns, P = 0.09; LPS-treated, TLR2-/- compared to WT: 1.17-fold decrease, ns, P = 0.63). [file ar4598-S3.jpeg]
