# Supplementary material for: Chronic Toll-like receptor 4 stimulation in skin induces inflammation, macrophage activation, transforming growth factor beta signature gene expression, and fibrosis
Source: Arthritis Res Ther. 2014 Jul 1;16(4):R136. doi: 10.1186/ar4598 (PMC4227089; doi:10.1186/ar4598)
Supplement: Additional file 2 — Pro-fibrotic gene expression in lipopolysaccharide (LPS)-treated skin. Pro-fibrotic gene expression in 1- and 4-week LPS-treated mice. In the second and third columns, it is reported as the fold-increase for each pro-fibrotic gene described in the first column, respectively in mice and in 4-week LPS-treated mice (Fold inc. 1-week and Fold inc. 4-week). In the last 2 columns, the P-values for each pro-fibrotic gene are reported (P-value 1-week and P-value 4-week). Fold-increase is calculated as the ratio of the mean of wild-type (WT) LPS-treated and WT PBS-treated mice gene expression. [file ar4598-S2.docx]

**Additional file 2. Pro-fibrotic gene expression in LPS-treated skin**

| **Pro-fibrotic genes** | **Fold-inc.1 w** | **Fold-inc.4 w** | **p value 1 w** | **p value 4 w** |
| --- | --- | --- | --- | --- |
| **Col5a1** | 2.11 | 2.64 | p<0.01 | P<0.05 |
| **LOX** | 3.27 | 4.98 | p<0.0001 | p<0.01 |
| **Mmp13** | 10.8 | 128.70 | P<0.0001 | p<0.01 |
| **Mmp3** | 5.65 | 8.17 | p<0.0001 | p<0.01 |
| **Sfrp2** | 6.23 | 15.29 | p<0.0001 | p<0.01 |
| **Wisp** | 3.4 | 8 | p<0.0001 | p<0.01 |
